# Supplementary material for: A New 1,3-Benzodioxole Compound from Hypecoum erectum and Its Antioxidant Activity
Source: Molecules. 2022 Oct 7;27(19):6657. doi: 10.3390/molecules27196657 (PMC9570887; doi:10.3390/molecules27196657)
Supplement: Supplementary file 1 [file molecules-27-06657-s001.zip › molecules-1922505-supplementary.pdf]

# Supplementary Information

## A New 1,3-Benzodioxole Compound from *Hypecoum erectum* and its Antioxidant Activity

Ning Xu [1,†],Wenli Bao[1,†], Jiletu Xin[1], Hua Xiao[1], Jiaqi Yu[1,*] and Liang Xu [1,*]

*1 Inner Mongolia Key Laboratory of the Natural Products Chemistry and Functional Molecular Synthesis, Inner Mongolia Minzu University, Tongliao 028000, China*

*Correspondence

† The authors contributed equally to this work

E-mail: xl405@imun.edu.cn (L. X.); jiaqiyu0126@163.com (J. Y.)

**Table of Contents**

**Figure S1.** $^{1}$H-NMR (500 MHz, $CDCl_3$-d) spectrum of compound **1**

**Figure S2.** $^{13}$C-NMR (125 MHz, $CDCl_3$-d) spectrum of compound **1**

**Figure S3.** HSQC spectrum of compound **1**

**Figure S4.** HMBC spectrum of compound **1**

**Figure S5.** $^{1}$H-$^{1}$HCOSY spectrum of compound **1**

**Figure S6.** HRESIMS spectrum of compound **1**

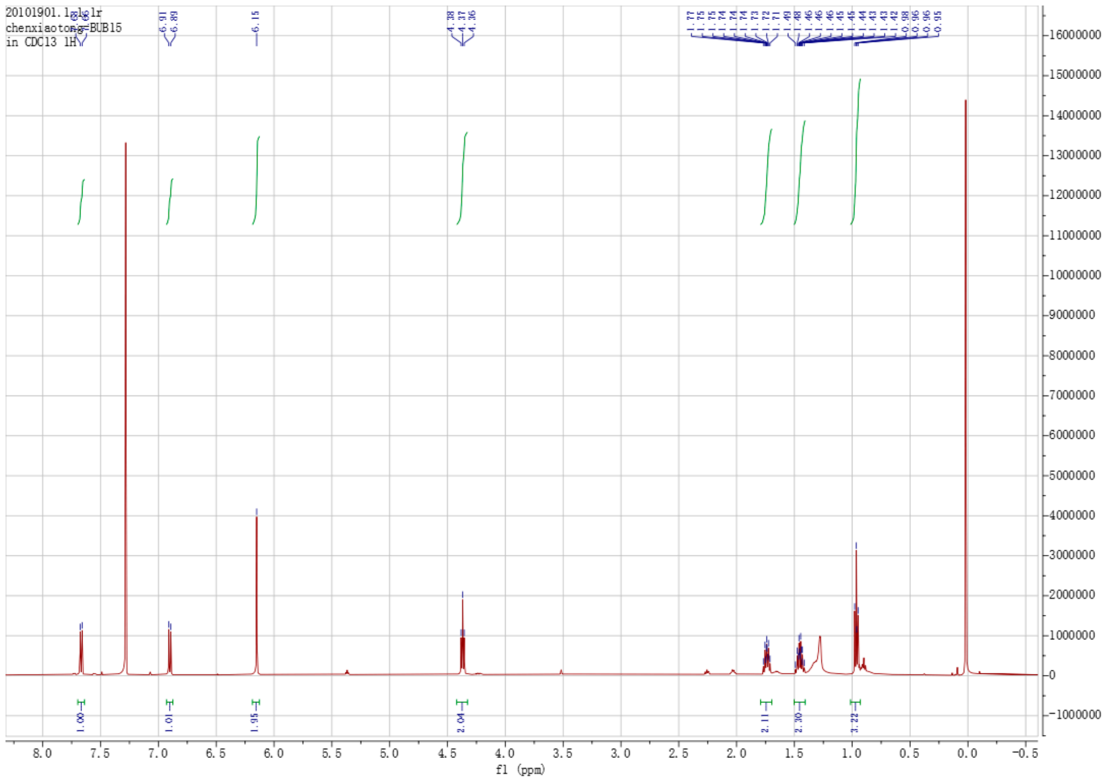


**Figure S1.** $^{1}$H-NMR (500 MHz, $CDCl_3$-d) spectrum of compound **1**

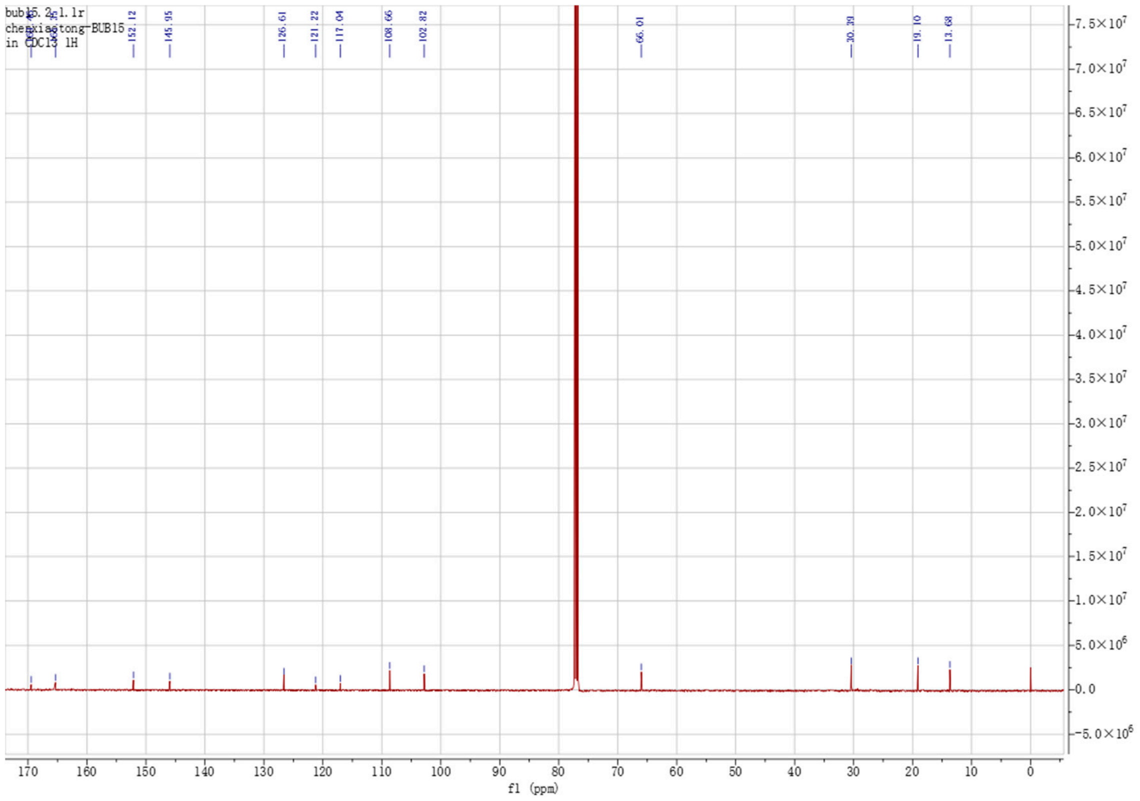


**Figure S2.** $^{13}$C-NMR (125 MHz, $CDCl_3$-d) spectrum of compound 1

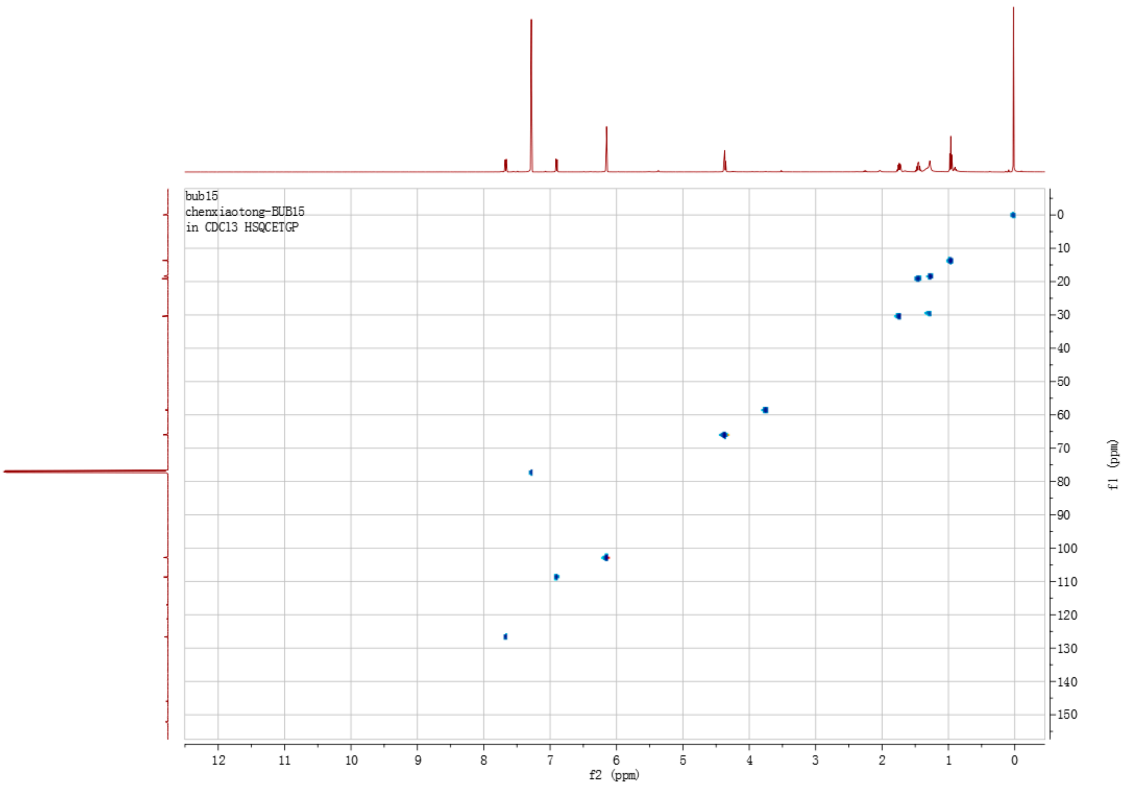


**Figure S3.** HSQC spectrum of compound **1**

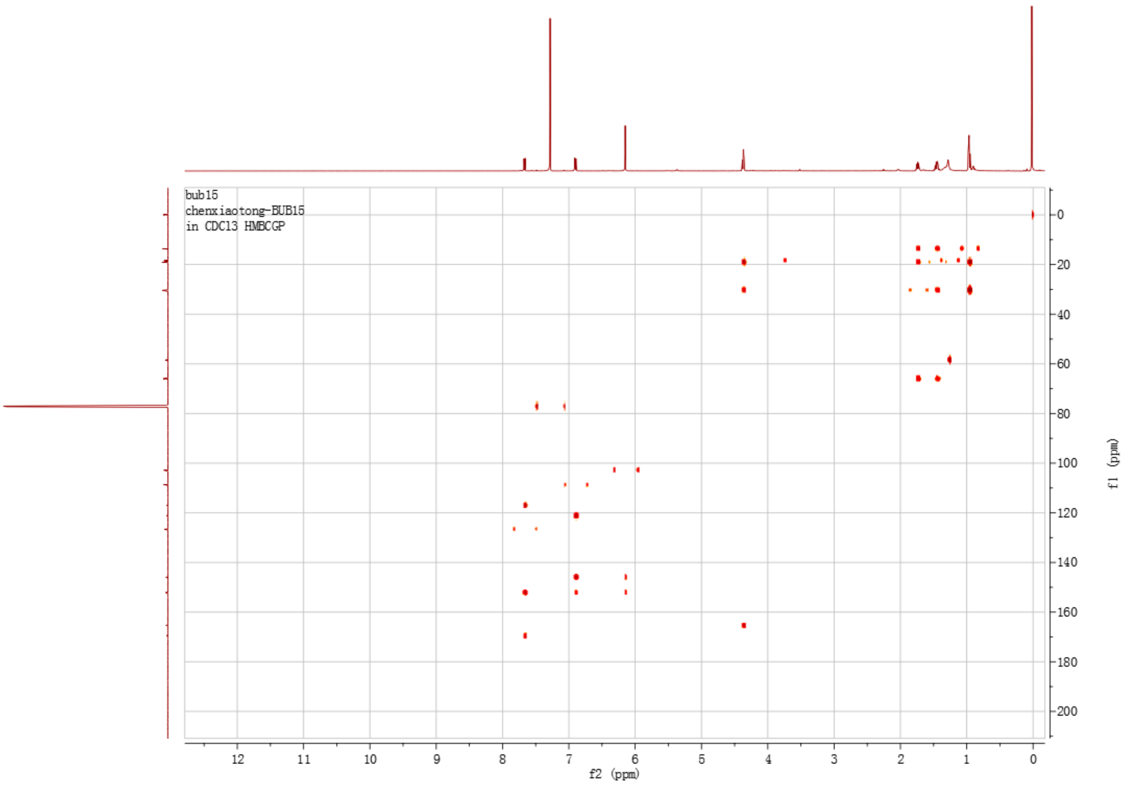


**Figure S4.** HMBC spectrum of compound **1**

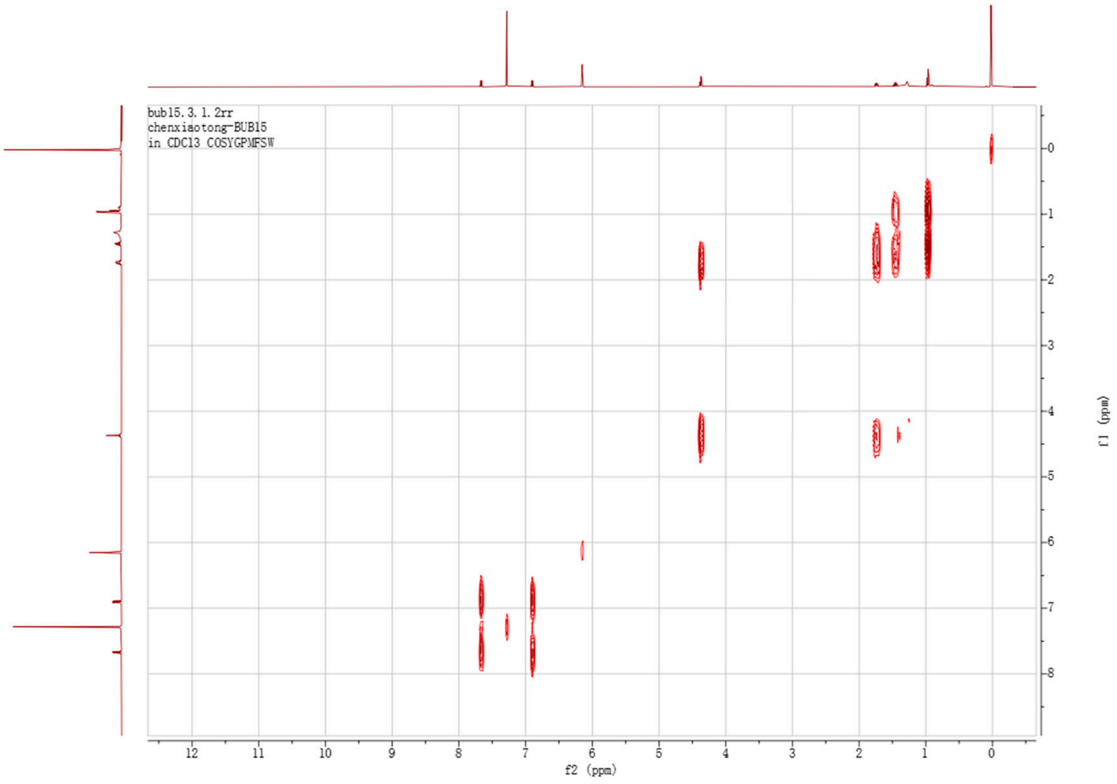


**Figure S5.** $^{1}H$-$^{1}H$COSY spectrum of compound **1**

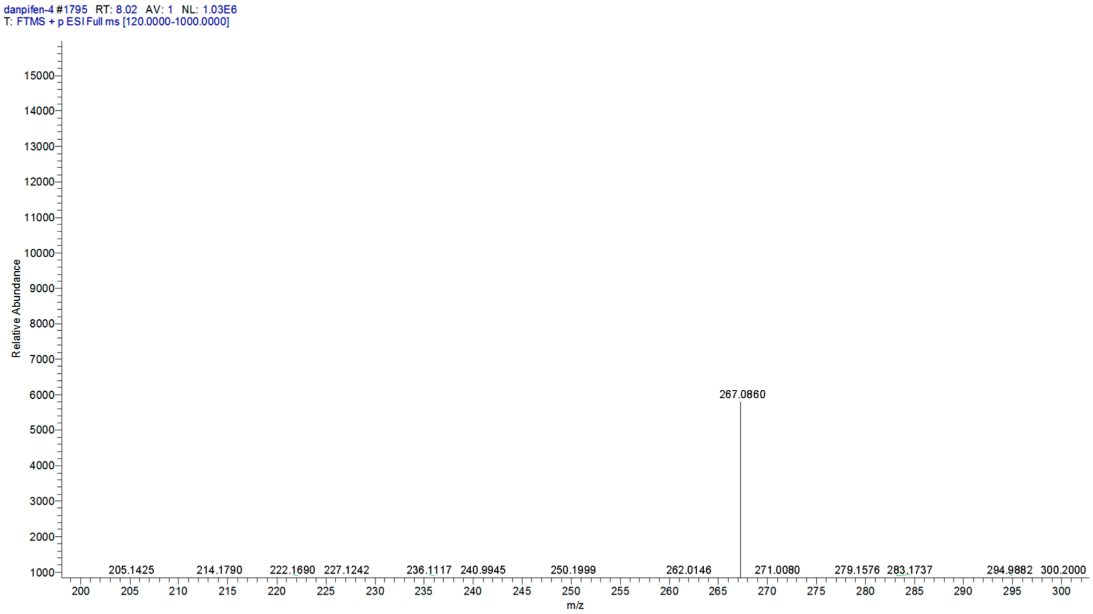


**Figure S6. HRESIMS spectrum of compound 1**
